# P-1394. Scenario Projections of Congenital Syphilis Cases in California, 2023–2030

**DOI:** 10.1093/ofid/ofae631.1570

**Published:** 2025-01-29

**Authors:** Natalie Linton, Nicole Burghardt, Natalie de Guzman, Robert Snyder, Tomás M León, Seema Jain, Kathleen Jacobson

**Affiliations:** California Department of Public Health, Richmond, California; California Department of Public Health, Richmond, California; California Department of Public Health, Richmond, California; California Department of Public Health, Richmond, California; California Department of Public Health, Richmond, California; California Department of Public Health, Richmond, California; CDPH, Richmond, California

## Abstract

**Background:**

The yearly number of congenital syphilis (CS) cases in California is at its highest point since 1950. We explored how efforts in California to 1) reduce syphilis cases; and 2) increase adequate syphilis treatment among infected pregnant persons could impact CS case projections through 2030.

Yearly projected incidence by scenario comparing the baseline scenario to a 10% reduction in infections, 10% increase in treatment adequacy, and both interventions combined

**Figure:**
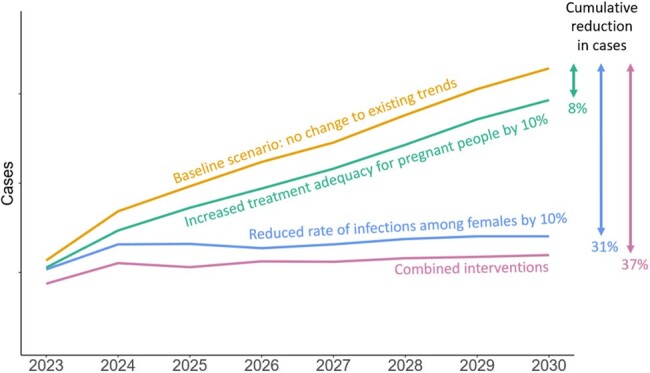

**Methods:**

We developed a scenario modeling framework to project CS incidence between 2023–2030 using California 2015–2022 surveillance data on CS cases and female syphilis cases of childbearing age (15–44 years). We compared a baseline scenario where existing trends continued without additional interventions to scenarios that considered 1) the impact of an absolute reduction of 2–10% in the rate of female syphilis cases per year; and 2) a 2–10% increase in the proportion of pregnant persons adequately treated for syphilis.

**Results:**

A reduction in the absolute rate of female syphilis cases by 2–10% led to an 8–31% decrease in cumulative CS cases between 2023–2030. Increasing the proportion of pregnant persons adequately treated for syphilis by 2–10% over baseline led to a 1–8% decrease in cumulative CS cases. Combining both scenarios resulted in an 8–37% reduction in cumulative cases.

**Conclusion:**

This model shows that to reduce CS case incidence in California, the most important upstream intervention is to reduce the rate of syphilis among females. To this end, syphilis screening and treatment among those who infect females must be improved. Additionally, even assuming no changes in syphilis incidence in females, CS cases could be reduced to a lesser extent by increasing the proportion of pregnant persons with syphilis who are adequately treated above current rates. The results of this study can inform syphilis screening and treatment recommendations, and the magnitude of future CS case reduction will depend on intervention scope and implementation.

**Disclosures:**

**All Authors**: No reported disclosures

